# ASA class is associated with early revision and reoperation after total hip arthroplasty: an analysis of the Geneva and Swedish Hip Arthroplasty Registries

**DOI:** 10.1080/17453674.2019.1605785

**Published:** 2019-04-30

**Authors:** Rory J Ferguson, Alan J Silman, Christophe Combescure, Erik Bulow, Daniel Odin, Didier Hannouche, Siôn Glyn-Jones, Ola Rolfson, Anne Lübbeke

**Affiliations:** a Nuffield Department of Orthopaedics, Rheumatology and Musculoskeletal Sciences, University of Oxford, UK;; b Division of Clinical Epidemiology, Geneva University Hospitals, Switzerland;; c The Swedish Hip Arthroplasty Register and the Department of Orthopaedics, Institute of Clinical Sciences, Sahlgrenska Academy, University of Gothenburg, Sweden;; d Division of Orthopaedics and Trauma Surgery, Geneva University Hospitals, Switzerland

## Abstract

Background and purpose — Data from several joint replacement registries suggest that the rate of early revision surgery after primary total hip arthroplasty (THA) is increasing. The ASA class, now widely recorded in arthroplasty registries, may predict early revision. We investigated the influence of ASA class on the risk of revision and other reoperation within 3 months and within 5 years of primary THA.

Patients and methods — We used data from the Geneva and Swedish Hip Arthroplasty Registries, on primary elective THAs performed in 1996–2016 and 2008–2016, respectively. 5,319 and 122,241 THAs were included, respectively. Outcomes were all-cause revision and other reoperations evaluated using Kaplan–Meier survival and Cox regression analyses.

Results — Within 3 months after surgery, higher ASA class was associated with greater risk of revision and other reoperation. 3-month cumulative incidences of revision by ASA class I, II, and III–IV respectively, were 0.6%, 0.7%, and 2.3% in Geneva and 0.5%, 0.8%, and 1.6% in Sweden. 3-month cumulative incidences of other reoperation were 0.4%, 0.7%, and 0.9% in Geneva and 0.2%, 0.4%, and 0.7% in Sweden. Adjusted hazard ratios (ASA III–IV vs. I) for revision within 3 months were 2.7 (95% CI 1.2–5.9) in Geneva and 3.3 (CI 2.6–4.0) in Sweden.

Interpretation — Assessment of ASA class of patients prior to THA will facilitate risk stratification. Targeted risk-reduction strategies may be appropriate during the very early postoperative period for patients identified as at higher risk. Systematically recording ASA class in arthroplasty registries will permit risk adjustment and facilitate comparison of revision rates internationally.

Data from several joint replacement registries suggest that the rate of early revision surgery after primary total hip arthroplasty (THA), widely defined as within 5 years of primary THA, is increasing (Thien et al. 2014, Cnudde et al. 2017). Recent data have shown that a high proportion of early revision surgeries are performed within 3 months (Swiss National Joint Registry 2018). Patients requiring such early revision surgery may share particular characteristics that put them at risk, such as preoperative health status.

Evidence from the New Zealand Joint Registry suggests that poor preoperative health status, assessed by ASA class, places patients at increased risk of revision within 2 years of surgery (Hooper et al. 2012). However, there are few data on the influence of poor preoperative health status on very early revision, specifically within 3 months of primary THA. Understanding its influence on the rate of very early revision surgery would be beneficial for 3 reasons: 1st, enhanced preoperative risk stratification would support surgeons and patients; 2nd, risk-reduction strategies could be identified and implemented for patients most at risk within 3 months postoperatively; and 3rd, risk adjustment would facilitate comparisons of outcomes between datasets.

Other reoperations after primary THA include, but are not limited to, debridement of infection, osteosynthesis of periprosthetic fracture, and drainage of hematoma. Data on other reoperations are not widely collected by arthroplasty registries. Evidence on incidence and causative factors is limited (Ferguson et al. 2018).

Many methods exist to measure preoperative health status. The ASA classification system is now the most widely collected system for measuring physical health status by arthroplasty registries worldwide (Lübbeke et al. 2018).

We investigated the influence of ASA class on the risk of revision and other reoperation within 3 months and within 5 years of primary THA. In cases of revision and other reoperation, we investigated the indication for surgery.  

## Patients and methods

We conducted a retrospective analysis of data from 2 arthroplasty registries. We performed a preliminary study in a hospital registry (Geneva Arthroplasty Registry, GAR), and compared the results with those from a national registry (Swedish Hip Arthroplasty Register, SHAR).

GAR collects data on all THAs performed at Geneva University Hospitals, the only public hospital of the canton of Geneva, Switzerland serving a population of 500,000 inhabitants (Geneva Joint Arthroplasty Registry 2017). Completeness of recording THAs is > 99%. SHAR collects data on all THAs performed in Sweden, covering 80 clinics (Swedish Hip Arthroplasty Register 2016). Completeness of recording THAs in the registry is 98.3%. The completeness of capture of revision surgery following primary THAs recorded in the GAR was 100% in 2013–2016, based on revisions performed within Switzerland. It was not possible to directly calculate the completeness of capture of revision surgery prior to 2013 in GAR; however, loss to follow-up in GAR after 5 years was 6% during 1996–2012, hence we estimate the completeness of capture of revision surgery was ≥ 94%. In SHAR the completeness of capture of revision surgery was 93%, based on revisions performed within Sweden.

Eligible procedures were elective primary THAs performed during the period that registries collected data on ASA class. This period was March 1996 through December 2016 for GAR and January 2008 through December 2016 for SHAR. THAs in patients with missing data on ASA class were excluded. Bilateral cases were included. 2 groups of cases were excluded: 1st, we excluded metal-on-metal THAs because they have a substantially higher revision rate than other bearings (Swedish Hip Arthroplasty Register 2016, Geneva Joint Arthroplasty Registry 2017, National Joint Registry 2017). Moreover, patients with lower ASA class received metal-on-metal prostheses more than other patients, meaning inclusion of such cases could have biased our results. 2nd, we excluded THAs for which the indication was trauma or malignancy.

The ASA classification system classifies patients into 6 categories (classes I [normal health]–VI [brain death]). ASA classes V and VI are not appropriate to patients undergoing elective THA, leaving a range of ASA classes I–IV.

We evaluated 2 outcomes: incidence of revision and of any other reoperation. Revision surgery was defined as any surgery that involved the addition, removal, or replacement of 1 or more components of the prosthetic hip. Other reoperation was defined as any surgery to the prosthetic hip that did not involve the addition, removal, or replacement of any components of the prosthetic hip. Closed reduction of dislocation was not included as a reoperation. Indications for surgery were also extracted. Covariates were age at surgery, sex, BMI, and diagnosis (primary or secondary osteoarthritis [OA]).

The GAR records revision, other reoperation, and mortality data continuously and actively follows patients up at 1, 5, 10, 15, and 20 years. The SHAR records revision and other reoperation data continuously. Mortality data are obtained from the Swedish Board of Health and Welfare. The end of follow-up was December 2016.

### Statistics

Analyses were conducted independently for each registry. Baseline characteristics were described using frequencies, proportions, means, and standard deviations (SDs). The proportion of THAs in patients of ASA class IV (0.6% in GAR and 0.4% in SHAR) was too small for meaningful analysis on its own. Thus, ASA was categorized into 3 groups: class I, II, and III–IV.

The cumulative mortality was assessed with Kaplan–Meier survival estimates. Cumulative incidence of revision and other reoperation by ASA class over 5-year follow-up after index THA was assessed using non-parametric models with death as competing event. As a sensitivity analysis, the survival analyses were re-run including only the 1st THA procedure in each patient. Cause-specific Cox proportional hazard models (presented as cause-specific hazard ratios [HRs] with 95% confidence intervals [CIs]) were used to assess the association between ASA class and risk of revision and other reoperation. Death was considered as a competing event. ASA class I was defined as the referent category. Details on the assumption of the models are presented in the Appendix (see Supplementary data). With the proposed models, the HRs for the associations were potentially different within 3 months following primary THA and after 3 months. Multivariable models with a pre-specified set of adjustment factors (age, sex, BMI, diagnosis) were conducted. Complete case analysis was used for adjusted models.

Data were analyzed using SPSS Version 23 software (IBM Corp, Armonk, NY, USA) and R (R Foundation for Statistical Computing, Vienna, Austria) with alpha of 0.05 as the statistical threshold for significance (all tests were 2-sided).

### Ethics, funding, and potential conflicts of interests

The registry data collection was approved by the Geneva University Hospital Institutional Review Board and the Gothenburg Regional Ethical Review Board. No funding was received for the study. The authors declare no potential conflicts of interest. 

## Results

In GAR, 5,319 procedures in 4,501 patients were eligible for inclusion. In SHAR, 122,241 procedures in 106,522 patients were eligible for inclusion (Table 1). In both cohorts the proportions of cases in obese patients (BMI ≥ 30), in those over 85 years of age, and in patients with secondary OA were highest in ASA classes III–IV.

**Table 1.** Preoperative patient characteristics by ASA class

	GAR				SHAR			
	ASA I	ASA II	ASA III–IV	Total	ASA I	ASA II	ASA III–IV	Total
	n = 481	n = 3,496	n = 1,342	n = 5,319	n = 29,280	n = 72,857	n = 20,104	n = 122,241
Women, n (%)	259 (54)	2,106 (60)	767 (57)	3,132 (59)	16,112 (55)	41,460 (59)	9,885 (53)	67,149 (57)
Age (mean, SD)	60.1 (12.3)	68.8 (11.7)	76.1 (10.1)	69.9 (12.2)	62.5 (10.7)	69.2 (9.7)	72.9 (9.8)	68.2 (10.5)
Age categories, n (%)								
< 55	150 (31)	393 (11)	42 (3.1)	585 (11)	6,325 (22)	5,152 (7.1)	863 (4.3)	12,340 (10)
55–64	127 (26)	667 (19)	109 (8.1)	903 (17)	9,679 (33)	15,756 (22)	2,701 (13)	28,136 (23)
65–74	152 (32)	1,226 (35)	357 (27)	1,735 (33)	9,766 (33)	29,660 (41)	6,999 (35)	46,425 (38)
75–84	49 (10)	1,024 (29)	550 (41)	1,623 (31)	3,225 (11)	19,423 (27)	7,648 (38)	30,296 (25)
**≥** 85	3 (0.6)	186 (5.3)	284 (21)	473 (8.9)	285 (1.0)	2,866 (3.9)	1,893 (9.4)	5,044 (4.1)
BMI (mean, SD)	24.8 (3.3)	26.8 (4.7)	27.3 (5.6)	26.8 (4.9)	26.1 (3.8)	27.4 (4.4)	28.7 (5.6)	27.3 (4.6)
BMI categories, n (%)								
< 18.5	13 (2.7)	63 (1.8)	43 (3.3)	119 (2.3)	195 (0.6)	531 (0.7)	240 (1.2)	966 (0.8)
18.5–24	245 (51)	1,280 (37)	437 (33)	1,962 (37)	11,405 (38)	20,933 (29)	5,008 (26)	37,346 (31)
25–29	193 (40)	1,303 (38)	441 (33)	1,937 (37)	12,868 (43)	31,374 (44)	6,955 (36)	51,197 (43)
30–34	27 (5.6)	642 (19)	288 (22)	957 (18)	3476 (12)	14,512 (20)	4,520 (23)	22,508 (19)
35–39	2 (0.4)	156 (4.5)	85 (6.4)	243 (4.6)	416 (1.3)	3,234 (4.5)	2,115 (11)	5,765 (4.8)
**≥** 40	0 (0.0)	26 (0.7)	27 (2.0)	53 (1.0)	66 (0.2)	529 (0.7)	629 (3.2)	1,224 (1.0)
Missing data	1	26	21	48	854	1,744	637	3,235
Diagnosis, n (%)								
Primary OA	375 (78)	2,817 (81)	1,007 (75)	4,199 (79)	26,644 (91)	67,221 (92)	17,666 (88)	111,531 (92)
Secondary OA	106 (22)	679 (19)	335 (25)	1,120 (21)	2,636 (9.0)	5,636 (7.7)	2,438 (12)	10,710 (8.8)

In GAR, 126 cases of revision were recorded within 5 years, with 59 (47% of total) within 3 months (Table 2). The incidence of death within 5 years was 12.8% (CI 11.8–13.8). In SHAR, 2,353 cases of revision were recorded within 5 years, with 1,030 (44% of total) within 3 months. The incidence of death within 5 years was 8.3% (CI 8.1–8.5). In both cohorts, the cumulative incidence of revision within 3 months and within 5 years was higher in ASA classes III–IV than in ASA class I (within 3 months, GAR: 2.3% versus 0.6%; SHAR: 1.6% versus 0.5%; within 5 years, GAR: 3.3% versus 2.3%; SHAR: 3.3% versus 1.9%). The cumulative incidence was lower for other reoperation than for revision in both cohorts.

**Table 2.** Incidence of revision and other reoperation within 5 years of primary THA by ASA score

	Total number	Revision Cumulative incidence (CI)		Total number	Other reoperation Cumulative incidence (CI)	
	(%)	3 months	5 years	(%)	3 months	5 years
Geneva						
All patients	126 (2.4)	1.1 (0.8–1.4)	2.6 (2.1–3.0)	95 (1.8)	0.7 (0.5–0.9)	1.9 (1.5–2.3)
ASA I	10 (2.1)	0.6 (0.0–1.3)	2.3 (0.9–3.8)	9 (1.9)	0.4 (0.0–1.0)	2.0 (0.7–3.4)
ASA II	73 (2.1)	0.7 (0.4–1.0)	2.3 (1.8–2.8)	58 (1.7)	0.7 (0.4–0.9)	1.8 (1.3–2.2)
ASA III–IV	43 (3.2)	2.3 (1.5–3.1)	3.3 (2.3–4.3)	28 (2.1)	0.9 (0.0 –1.4)	2.2 (1.4–3.0)
Sweden						
All patients	2,353 (1.9)	0.9 (0.8–0.9)	2.3 (2.2–2.4)	878 (0.7)	0.4 (0.4–0.4)	0.8 (0.8–0.9)
ASA I	444 (1.5)	0.5 (0.4–0.6)	1.9 (1.7–2.1)	145 (0.5)	0.2 (0.2–0.3)	0.6 (0.5–0.7)
ASA II	1,364 (1.9)	0.8 (0.7–0.9)	2.3 (2.2–2.4)	522 (0.7)	0.4 (0.3–0.4)	0.8 (0.8–0.9)
ASA III–IV	545 (2.7)	1.6 (1.4–1.7)	3.3 (3.0–3.6)	211 (1.0)	0.7 (0.6–0.8)	1.2 (1.0–1.4)

There was a positive association between ASA class and the risk of revision within 5 years (GAR: p = 0.02 for the comparison between ASA class I or II versus III or IV; SHAR: p < 0.001 for the comparison among all ASA classes). Results were unchanged by including only the first procedure in each patient. An association between ASA class and risk of other reoperation within 5 years was detected only in SHAR (GAR: p = 0.6; SHAR: p < 0.001) (Figure).

In GAR, ASA classes III–IV were associated with a higher risk of revision (Table 3). However, the association was restricted to within 3 months after primary THA (unadjusted HR: 3.4, CI 1.6–7.4). The association persisted after adjustment for differences in the preoperative baseline characteristics. The risk of revision was also higher in patients with a diagnosis of secondary OA and in obese patients.

**Table 3.** Associations with the risk of revision (time-invariant HR unless specified)

	GAR cohort	SHAR cohort
Model	HR (CI)	HR (CI)
Univariable model		
ASA I	1 (ref)	1 (ref)
ASA II	1.0 (0.5–2.0)	1.6 (1.4–2.0) **^a,f^**
		1.1 (0.9–1.3) **^b,f^**
ASA III–IV	3.4 (1.6–7.4) **^a,c^**	3.2 (2.6–3.9) **^a,f^**
	0.7 (0.3–1.7) **^b,c^**	1.3 (1.1–1.6) **^b,f^**
Multivariable model		
ASA I	1 (ref)	1 (ref)
ASA II	1.0 (0.5–1.9)	1.7 (1.4–2.1) **^a,g^**
		1.2 (1.0–1.3) **^b,g^**
ASA III–IV	2.7 (1.2–5.9) **^a,d^**	3.3 (2.6–4.0) **^a,g^**
	0.7 (0.3–1.7) **^b,d^**	1.4 (1.1–1.6) **^b,g^**
Sex		
Male	1 (ref)	1 (ref)
Female	0.8 (0.6–1.1)	0.7 (0.6–0.7)
Diagnosis		
Primary OA	1 (ref)	1 (ref)
Secondary OA	2.4 (1.7–3.5)	1.4 (1.3–1.6)
BMI		
< 35	1 (ref)	1 (ref)
**≥** 35	3.7 (2.3–6.0)	2.6 (2.2–3.1) **^a,h^**
		1.2 (1.0–1.5) **^b,h^**
Age		
< 85 y	1 (ref)	1 (ref)
**≥** 85 y	1.6 (0.8–3.3) **^a,e^**	1.9 (1.5–2.4) **^a,i^**
	0.2 (0.1–1.7) **^b,e^**	0.6 (0.4–0.8) **^b,i^**

**^a^**HR within the first 3 months.

**^b^**HR after 3 months and within 5 years.

**^c^**The change in HR within the first 3 months and after was statistically significant (p < 0.001).

**^d^**The change in HR within the first 3 months and after was statistically significant (p = 0.002).

**^e^**A change in HR within the first 3 months and after was suspected (p = 0.07).

**^f^**The change in HR within the first 3 months and after was statistically significant (ASA II: p < 0.001, ASA III–IV: p < 0.001).

**^g^**The change in HR within the first 3 months and after was statistically significant (ASA II: p < 0.001, ASA III–IV: p < 0.001).

**^h^**The change in HR within the first 3 months and after was statistically significant (p < 0.001).

**^i^**The change in HR within the first 3 months and after was statistically significant (p < 0.001).

In SHAR, the association of ASA classes III–IV with revision within 3 months was confirmed (unadjusted HR: 3.2, CI 2.6–3.9). The association decreased after 3 months but remained statistically significant (unadjusted HR: 1.3, CI 1.1–1.6). In contrast to GAR, an association with ASA class II was also detected within 3 months (unadjusted HR: 1.6, CI 1.4–2.0). Adjustment for differences in the preoperative baseline characteristics did not importantly modify the associations. In contrast to GAR, sex and age were also associated with the risk of revision. Women had a lower risk of revision. Patients aged over 85 years had a higher risk within 3 months but a lower risk thereafter. The associations between the risk of revision and the diagnosis of both secondary OA and obesity were confirmed in SHAR.

In GAR, ASA class was not associated with the risk of other reoperation (unadjusted HR ASA III–IV vs. ASA I: 1.2, CI 0.6–2.5) (Table 4, see Supplementary data). In SHAR, ASA class was associated with a greater risk of other reoperation within 3 months following primary THA than after 3 months and within 5 years (unadjusted HR ASA III–IV vs. ASA I within 3 months: 3.2, CI 2.3–4.3; after 3 months and within 5 years: 1.6, CI 1.2–2.1).

**Table 4.** Associations with the risk of other reoperation (time-invariant HR unless specified)

	GAR cohort	SHAR cohort
Model	HR (CI)	HR (CI)
Univariable model		
ASA I	1 (ref)	1 (ref)
ASA II	0.9 (0.5–1.8)	1.8 (1.3–2.4)^a^**^,f^**
		1.2 (1.0–1.6)^b^**^,f^**
ASA III–IV	1.2 (0.6–2.5)	3.2 (2.3–4.3)^a^**^,f^**
		1.6 (1.2–2.1)^b^**^,f^**
Multivariable model		
ASA I	1 (ref)	1 (ref)
ASA II	0.9 (0.4–1.7)	1.7 (1.2–2.2)^a^**^,g^**
		1.1 (0.9–1.5)^b^**^,g^**
ASA III–IV	1.1 (0.5–2.4)	2.7 (2.0–3.7)^a^**^,g^**
		1.4 (1.0–1.8)^b^**^,g^**
Sex		
Male	1 (ref)	1 (ref)
Female	0.8 (0.4–1.6)^a^**^,c^**	0.6 (0.5–0.8)^a^**^,h^**
	1.8 (1.0–3.2)^b^**^,c^**	1.0 (0.8–1.2)^b^**^,h^**
Diagnosis		
Primary OA	1 (ref)	1 (ref)
Secondary OA	3.1 (1.6–6.0)^a^**^,d^**	1.8 (1.5–2.2)
	0.7 (0.4–1.5)^b^**^,d^**	
BMI		
< 35	1 (ref)	1 (ref)
**≥** 35	4.5 (1.9–10.3)^a^**^,e^**	2.6 (1.9–3.4)^a^**^,i^**
	1.1 (0.4–2.9)^b^**^,e^**	1.2 (0.8–1.7)^b^**^,i^**
Age		
< 85 y	1 (ref)	1 (ref)
**≥** 85 y	0.9 (0.4–2.0)	1.9 (1.4–2.4)

^a^HR within the first 3 months.

^b^HR after 3 months and within 5 years.

^c^A change in HR within the first 3 months and after was suspected (p = 0.08).

^d^The change in HR within the first 3 months and after was statisti-cally significant (p = 0.003).

^e^A change in HR within the first 3 months and after was suspected (p = 0.05).

^f^The change in HR within the first 3 months and after was statisti-cally significant (ASA II: p = 0.05, ASA III–IV: p = 0.001).

^g^The change in HR within the first 3 months and after was statisti-cally significant for ASA II (p = 0.05) and close to statistical signifi-cance for ASA III–IV (p = 0.05).

^h^The change in HR within the first 3 months and after was statisti-cally significant (p < 0.001).

^i^The change in HR within the first 3 months and after was statisti-cally significant (p = 0.001).

The most frequent indications for revision in both cohorts were dislocation, infection, and periprosthetic fracture, and for other reoperation in both cohorts were infection, periprosthetic fracture, and hematoma (Table 5). 

**Table 5.** Indications for revision and other reoperation within 3 months by ASA grade

	Geneva				Sweden			
	ASA I	ASA II	ASA III–IV	Total	ASA I	ASA II	ASA III–IV	Total
Factor	n = 481	n = 3,496	n = 1,342	n = 5,319	n = 29,280	n = 72,857	n = 20,104	n = 122,241
Indications for revision, n (%)								
Dislocation	1 (0.2)	5 (0.1)	12 (0.9)	18 (0.3)	30 (0.1)	103 (0.1)	57 (0.3)	190 (0.2)
Infection	1 (0.2)	8 (0.2)	7 (0.5)	16 (0.3)	81 (0.3)	356 (0.5)	195 (1)	632 (0.5)
Periprosthetic fracture	0	6 (0.2)	7 (0.5)	13 (0.2)	24 (0.1)	80 (0.1)	42 (0.2)	146 (0.1)
Aseptic loosening	0	4 (0.1)	3 (0.2)	7 (0.1)	8 (< 0.1)	21 (< 0.1)	8 (< 0.1)	37 (< 0.1)
Implant malposition	0	1 (< 0.1)	1 (0.1)	2 (< 0.1)	3 (< 0.1)	8 (< 0.1)	3 (< 0.1)	14 (< 0.1)
Other ^a^	1 (0.2)	1 (< 0.1)	1 (0.1)	3 (0.1)	1 (< 0.1)	2 (< 0.1)	3 (< 0.1)	6 (< 0.1)
Unknown	0	0	0	0	2 (< 0.1)	3 (< 0.1)	0	5 (< 0.1)
Total	3 (0.6)	25 (0.7)	31 (2.3)	59 (1.1)	149 (0.5)	573 (0.8)	308 (1.5)	1,030 (0.8)
Indications for reoperation, n (%)								
Infection	1 (0.2)	11 (0.3)	3 (0.2)	15 (0.3)	45 (0.2)	219 (0.3)	106 (0.5)	370 (0.3)
Periprosthetic fracture	0	3 (0.1)	6 (0.4)	9 (0.2)	4 (< 0.1)	16 (< 0.1)	10 (< 0.1)	30 (< 0.1)
Hematoma	1 (0.2)	4 (0.1)	1 (0.1)	6 (0.1)	1 (< 0.1)	9 (< 0.1)	8 (< 0.1)	18 (< 0.1)
Abductor avulsion	0	2 (0.1)	1 (0.1)	3 (0.1)	1 (< 0.1)	1 (< 0.1)	0	2 (< 0.1)
Dislocation	0	0	0	0	3 (< 0.1)	8 (< 0.1)	1 (< 0.1)	12 (< 0.1)
Cement problem	0	0	0	0	1 (< 0.1)	3 (< 0.1)	2 (< 0.1)	6 (< 0.1)
Other ^b^	0	3 (0.1)	1 (0.1)	4 (0.1)	2 (< 0.1)	7 (< 0.1)	1 (< 0.1)	10 (< 0.1)
Unknown	0	0	0	0	1 (< 0.1)	3 (< 0.1)	2 (< 0.1)	6 (< 0.1)
Total	2 (0.4)	23 (0.7)	12 (0.9)	37 (0.7)	58 (0.2)	266 (0.4)	130 (0.6)	454 (0.4)

**^a^**Other includes: infection suspected but not confirmed; hematoma; other material left in joint; nerve injury; delayed healing; pain.

**^b^**Other includes: infection suspected but not confirmed; pain; allergy to suture; other material left in joint; aseptic loosening.

## Discussion

Our study had 3 important findings on outcomes within 3 months after primary THA. 1st, preoperative ASA classes III–IV compared with ASA class I were associated with a more than 3 times higher risk of very early revision. 2nd, preoperative ASA classes III–IV compared with ASA class I were associated with a more than 2 times higher risk of very early other reoperation in SHAR. These risks were independent of age, sex, BMI, and diagnosis. 3rd, a substantial proportion of early revision and other reoperation procedures in patients with ASA classes III–IV were performed within 3 months of primary THA. We also found that beyond 3 months and within 5 years after primary THA patients with increased ASA class were not at increased risk of revision in GAR and were only at a slightly higher risk of revision in SHAR.

This study has limitations. 1st, the ASA classification system has been criticized because of the subjective nature of the assessment, which has poor inter-observer correlation (Ranta et al. 1997, Mak et al. 2002). Despite this, as noted by Hooper et al. (2012), the ASA classification system has remained the most widely used anesthetic preoperative assessment and the most widely collected tool for measuring comorbidity by arthroplasty registries worldwide. We accept that there may be poor inter-observer reliability when determining between ASA class I and II, but we agree with Hooper et al. that the difference between ASA class I and III is so profound (a normal healthy patient compared with a patient with severe systemic disease) that we believe that the significance of our results, when comparing ASA class I with ASA classes III–IV, was unlikely to be affected by this potential error.

2nd, the ASA class in our data represents only a snapshot of the physical health status of each patient, taken immediately prior to primary THA. This is a general drawback of using ASA class because it is assessed only in the context of surgery. We do not know whether physical health status changed subsequent to THA, and if this had an influence on revision and other reoperation rates. With increasing follow-up, other health changes might intervene that would attenuate the influence of a single baseline measure. However, we aimed to determine the influence of preoperative physical health status, to enable the identification of high-risk patients preoperatively, so the possibility of subsequent changes should not detract from our results.

3rd, our results were adjusted for age, sex, BMI, and diagnosis, but other factors may have had a confounding effect. Here, we elected to focus on patient factors that are routinely measured before THA, and so may be used when counselling patients considering THA. We did not adjust for surgical factors, such as surgical approach and implant fixation, which are known also to influence early revision and other reoperation rates (Jämsen et al. 2014, Meneghini et al. 2017). The reason for this decision is that each of these factors is chosen by the surgeon, and these choices may be influenced by ASA class, age, and BMI. Therefore, the inclusion of surgical factors in the models may lead to over-adjustment.

We included both procedures in patients who had undergone bilateral THA. This is because the ASA class may change between the 2 operations. Indeed, a sensitivity analysis in GAR and SHAR including only the first procedure in each patient showed similar results to the analyses, including bilateral cases. Nevertheless, since ASA class is associated with outcome, the preoperative ASA class of the 2nd THA would influence the outcome of both the 1st and 2nd THAs. Thus, a degree of correlation in patients with bilateral THAs cannot be excluded.

4th, GAR is a small registry and we included cases since 1996. This might limit the applicability to modern patients; however, we compared the results with results in the larger SHAR with recent data and the results were similar.

Many studies have suggested that patients with poor overall physical health have a higher risk of early revision. A systematic review found 5 papers that reported greater preoperative comorbidity was associated with a higher risk of revision, with outcome ranging from 6 months to 8 years (Prokopetz et al. 2012). The Charlson Comorbidity Index was used as the measure of comorbidity in these papers. Whilst the Charlson score is widely validated, its limitation is that it simply considers the presence or absence of certain diseases and does not account for their severity. Furthermore, it requires more information than the ASA classification system to complete and is not routinely calculated before THA, or widely collected by registries.

To our knowledge, 2 reports of arthroplasty registry data have investigated the influence of ASA class on early revision rate. Hooper et al. (2012) found an adjusted hazard ratio of 1.4 (CI 1.0–2.0) when comparing revision rates of patients with ASA class I versus III within 2 years postoperatively in the New Zealand Joint Registry. This agrees with our findings; however, the effect of increased ASA class on revision rate was less than in our study. This difference may be because Hooper et al. studied the longer period of 2 years postoperatively. Our data indicate that the effect of increased ASA class is highest within 3 months and decreases thereafter. The 2018 annual report of the Dutch Arthroplasty Registry reported graphically the revision rate after primary THA stratified by ASA class (Dutch Arthroplasty Register 2018). Although numerical data are not reported, the survival curve demonstrates a higher revision rate within 3 months of primary THA in ASA classes III–IV cases than ASA class I cases, similar to the trend observed in GAR and SHAR.

Data on the rate of other reoperations are scarce and we could find no previous study that had investigated the influence of preoperative health status on other reoperation rate with which to compare our results. This likely reflects several factors. 1st, the previous lack of a formal definition of what constitutes reoperation after arthroplasty. 2nd, study of revisions has taken precedence because they are seen as a more serious complication. 3rd, very few arthroplasty registries collect data on other reoperations.

Our study is the first to demonstrate that poor preoperative physical health status as measured with the ASA class is associated with increased risk of early other reoperation. We note the cumulative incidence of other reoperation was higher in GAR. Whilst there may be a real difference in other reoperation rate between the 2 registries, this observed difference alternatively may reflect greater completeness of capture of other reoperations in GAR.

That a substantial proportion of early revision and other reoperation procedures, performed in patients with ASA classes III–IV, occurs within 3 months of primary THA is a clinically important finding. It identifies this period of 3 months as critical to efforts to reduce revision and other reoperation rates. Infection, periprosthetic fracture, and dislocation were the most frequent indications for very early revision and other reoperation. Strategies to reduce the risk of revision targeted to these complications during this very early postoperative period may be designed and implemented for patients with ASA classes III–IV most at risk, and are a key focus for future work. These may include preoperative, perioperative, and postoperative interventions. Such intensive strategies may be appropriate and acceptable in this cohort of patients over this time frame with regards to patient preference and resource constraints.

In summary, our study has identified that within 3 months of primary elective THA patients with preoperative ASA classes III–IV have a higher risk of revision and other reoperation. The proposed benefits are improved patient counselling, targeted risk-reduction strategies, and improved risk adjustment between datasets.

### Supplementary data

Table 4 and Appendix are available as supplementary data in the online version of this article, http://dx.doi.org/10.1080/ 17453674.2019.1605785

RJF, AJS, OR, and AL contributed to the conception and design of the study. RJF, CC, EB, and DO performed the statistical analyses. RJF, AJS, and AL drafted the manuscript. All authors critically revised the manuscript.

The authors would like to thank all GAR and SHAR registry staff, and surgeons and patients who have contributed to the registries.

*Acta* thanks Marianne Hansen Gillam and Liza N van Steenbergen for help with peer review of this study.

**Figure:**
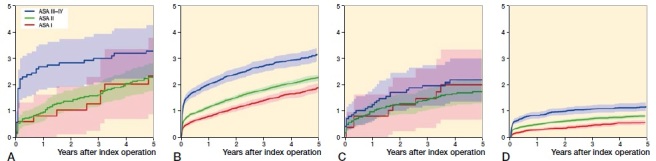
Cumulative incidence of revision in (A) GAR and (B) SHAR and cumulative incidence of other reoperation in (C) GAR and (D) SHAR by ASA class (95% CI shown in shading). The association with ASA class was statistically significant in SHAR (p < 0.001 for both revision and other reoperation). In GAR, the p-value was 0.07 for revision but the difference was statistically significant between ASA class III or IV and ASA class I or II (p = 0.02). No association with ASA class was detected in GAR for other reoperation (p = 0.6).

## Supplementary Material

Supplemental Material

## References

[CIT0001] Cnudde P, Nemes S, Bülow E, Timperley J, Malchau H, Kärrholm J, Garellick G, Rolfson O Trends in hip replacements between 1999 and 2012 in Sweden. J Orthop Res 2017; 36(1): 432–42. 28845900 10.1002/jor.23711 PMC5873269

[CIT0002] Dutch Arthroplasty Register Landelijke Registratie Orthopedische Implantaten Annual Report 2018.

[CIT0003] Ferguson R J, Palmer A J, Taylor A, Porter M L, Malchau H, Glyn-Jones S Hip replacement. Lancet 2018; 392(10158): 1662–71. 30496081 10.1016/S0140-6736(18)31777-X

[CIT0004] Geneva Joint Arthroplasty Registry Geneva Joint Arthroplasty Registry: Annual Report 2017. Available on request: christophe.barea@hcuge.ch.

[CIT0005] Hooper G J, Rothwell A G, Hooper N M, Frampton C The relationship between the American Society of Anesthesiologists physical rating and outcome following total hip and knee arthroplasty: an analysis of the New Zealand Joint Registry. J Bone Joint Surg Am 2012; 94(12): 1065–70. 22717825 10.2106/JBJS.J.01681

[CIT0006] Jämsen E, Eskelinen A, Peltola M, Mäkelä K High early failure rate after cementless hip replacement in the octogenarian. Clin Orthop Relat Res 2014; 472(9): 2779–89. 24771260 10.1007/s11999-014-3641-7 PMC4117887

[CIT0007] Lübbeke A, Silman A J, Barea C, Prieto-Alhambra D, Carr A J Mapping existing hip and knee replacement registries in Europe. Health Policy (New York) 2018; 122(5): 548–57. 10.1016/j.healthpol.2018.03.010 29598886

[CIT0008] Mak P H K, Campbell R C H, Irwin M G, American Society of Anesthesiologists The ASA physical status classification: inter-observer consistency. American Society of Anesthesiologists. Anaesth Intensive Care 2002; 30(5): 633–40. 12413266 10.1177/0310057X0203000516

[CIT0009] Meneghini R M, Elston A S, Chen A F, Kheir M M, Fehring T K, Springer B D Direct anterior approach: risk factor for early femoral failure of cementless total hip arthroplasty. J Bone Joint Surg 2017; 99(2): 99–105. 28099299 10.2106/JBJS.16.00060

[CIT0010] National Joint Registry National Joint Registry for England, Wales, Northern Ireland and the Isle of Man: 14th Annual Report 2017.

[CIT0011] Prokopetz J J, Losina E, Bliss R L, Wright J, Baron J A, Katz J N Risk factors for revision of primary total hip arthroplasty: a systematic review. BMC Musculoskelet Disord 2012; 13: 251. 23241396 10.1186/1471-2474-13-251 PMC3541060

[CIT0012] Ranta S, Hynynen M, Tammisto T A survey of the ASA physical status classification: significant variation in allocation among Finnish anaesthesiologists. Acta Anaesthesiol Scand 1997; 41(5): 629–32. 9181166 10.1111/j.1399-6576.1997.tb04755.x

[CIT0013] Swedish Hip Arthroplasty Register The Swedish Hip Arthroplasty Register: Annual Report 2016.

[CIT0014] Swiss National Joint Registry Swiss National Joint Registry: Annual Report 2018.

[CIT0015] Thien T M, Chatziagorou G, Garellick G, Furnes O, Havelin L I, Mäkelä K, Overgaard S, Pedersen A, Eskelinen A, Pulkkinen P, Kärrholm J Periprosthetic femoral fracture within two years after total hip replacement: analysis of 437,629 operations in the Nordic Arthroplasty Register Association database. J Bone Joint Surg Am 2014; 96(19): e167. 25274795 10.2106/JBJS.M.00643

