# Supplementary material for: ASA class is associated with early revision and reoperation after total hip arthroplasty: an analysis of the Geneva and Swedish Hip Arthroplasty Registries
Source: Acta Orthop. 2019 Apr 30;90(4):324–30. doi: 10.1080/17453674.2019.1605785 (PMC6718172; doi:10.1080/17453674.2019.1605785)
Supplement: Supplemental Material [file IORT_A_1605785_SM0444.pdf]

Supplementary data

Table 4. Associations with the risk of other reoperation (time-invariant HR unless specified)

Model	GAR cohort HR (CI)	SHAR cohort HR (CI)
Univariable model		
ASA I	1 (ref)	1 (ref)
ASA II	0.9 (0.5–1.8)	1.8 (1.3–2.4) [a,f]
		1.2 (1.0–1.6) [b,f]
ASA III–IV	1.2 (0.6–2.5)	3.2 (2.3–4.3) [a,f]
		1.6 (1.2–2.1) [b,f]
Multivariable model		
ASA I	1 (ref)	1 (ref)
ASA II	0.9 (0.4–1.7)	1.7 (1.2–2.2) [a,g]
		1.1 (0.9–1.5) [b,g]
ASA III–IV	1.1 (0.5–2.4)	2.7 (2.0–3.7) [a,g]
		1.4 (1.0–1.8) [b,g]
Sex		
Male	1 (ref)	1 (ref)
Female	0.8 (0.4–1.6) [a,c]	0.6 (0.5–0.8) [a,h]
	1.8 (1.0–3.2) [b,c]	1.0 (0.8–1.2) [b,h]
Diagnosis		
Primary OA	1 (ref)	1 (ref)
Secondary OA	3.1 (1.6–6.0) [a,d]	1.8 (1.5–2.2)
	0.7 (0.4–1.5) [b,d]	
BMI		
< 35	1 (ref)	1 (ref)
≥ 35	4.5 (1.9–10.3) [a,e]	2.6 (1.9–3.4) [a,i]
	1.1 (0.4–2.9) [b,e]	1.2 (0.8–1.7) [b,i]
Age		
< 85 y	1 (ref)	1 (ref)
≥ 85 y	0.9 (0.4–2.0)	1.9 (1.4–2.4)

[a] HR within the first 3 months.
[b] HR after 3 months and within 5 years.
[c] A change in HR within the first 3 months and after was suspected ($p = 0.08$).
[d] The change in HR within the first 3 months and after was statistically significant ($p = 0.003$).
[e] A change in HR within the first 3 months and after was suspected ($p = 0.05$).
[f] The change in HR within the first 3 months and after was statistically significant (ASA II: $p = 0.05$, ASA III–IV: $p = 0.001$).
[g] The change in HR within the first 3 months and after was statistically significant for ASA II ($p = 0.05$) and close to statistical significance for ASA III–IV ($p = 0.05$).
[h] The change in HR within the first 3 months and after was statistically significant ($p < 0.001$).
[i] The change in HR within the first 3 months and after was statistically significant ($p = 0.001$).

Appendix

Checking of models

The proportionality of hazards was checked by plotting the complementary log-log of Kaplan-Meier's survival (with censoring death) versus the log of the follow-up time. In addition, for each covariate (gender, age, BMI, diagnosis), univariate cause-specific Cox models were used to detect a variation of HRs within 3 months following primary THA and after 3 months. In cases where the proportional hazards assumption over follow-up time was violated, a time-varying HR was introduced in the model using a time-dependent variable and an interaction term.

Statistical method for the assessment of cause-specific Cox models

For the analyses of the risk of revision, we ran a cause-specific Cox model with death as competing risk and with an interaction to allow a variation of the hazard ratio over follow-up time. For this purpose, we used the function coxph of R software (package Survival). Data were organized as follow:

IdPatient	TimeStart (days)	TimeStop (days)	Event	ASA 2	ASA 3/4	Group Time
1	0	90	0	No	Yes	0
1	90	540	1	No	Yes	1
2	0	90	0	Yes	No	0
2	90	120	2	Yes	No	1
…						

The coding of the variable "Event" was: 0=censor, 1=revision, 2=death.

If a patient was censored or had a revision or died after 90 days, data were stored on two lines: one per period (first 3 months and after 3 months). The variable "GroupTime" indicated the period of time. The struture of database was consistent with the documentation of the R package Survival (see Therneau et al. https://cran.r-project.org/web/packages/survival/vignettes/timedep.pdf).

In Cox model, the hazard function for revision, hRevision (t), was modelled as follow:

$$h_{Revision}(t) = h_{Revision,0}(t) \exp[\ \alpha\ I_{(ASA = 2)} + \beta\ I_{(ASA = 2)}\ GroupTime + \gamma\ I_{(ASA = 3/4)} + \delta\ I_{(ASA = 3/4)}\ GroupTime]$$

were $h_0(t)$ is the baseline hazard of revision, α is the coefficient of ASA class = 2 (with ASA class = 1 as reference category), β is the coefficient of the interaction between ASA class = 2 and the period of time, γ is the coefficient of ASA class = 3 or 4 and δ is the coefficient of the interaction between ASA class = 3/4 and the period of time. With this model, the cause

specific hazard ratio for the association between ASA class 2 and revision was exp(α) in the first 3 months and exp($\alpha + \beta$) after 3 months. The test of the null hypothesis $\beta = 0$ allowed testing that the hypothesis that the association between ASA class = 2 and revision was the same in the two periods of time. Similarily, the cause specific hazard ratio for the association between ASA class 3 or 4 and revision was exp(γ) in the first 3 months and exp($\gamma + \delta$) after 3 months.

The code written in software R to assess the regression coefficients was:

```
coxph(Surv(TimeStart,TimeStop, Event==1) ~ ASAClass2+ ASAClass2:GroupTime+ ASAClass3+ ASAClass3:GroupTime)
```

For the additional models (with adjustment for sex, age and BMI and for the risk of other reoperation), a similar approach was followed.
